# Feasibility and preliminary effects of a theory-based self-management program for kidney transplant recipients: A pilot study

**DOI:** 10.1371/journal.pone.0248947

**Published:** 2021-06-30

**Authors:** Hye Won Jeong, Chi Eun Song, Minjeong An

**Affiliations:** 1 Department of Nursing, Chonnam National University Hospital, Gwangju, South Korea; 2 Department of Nursing, Nambu University, Gwangju, South Korea; 3 Interdisciplinary Program of Arts & Design Technology, College of Nursing, Chonnam National University, Gwangju, South Korea; University of Bristol, UNITED KINGDOM

## Abstract

Self-care activities are important to prevent transplant-related side effects and complications among kidney transplant recipients. Therefore, we developed a theory-based self-management program for kidney transplant recipients hospitalized after surgery. This study aimed to examine the feasibility of the program and to identify its preliminary effects on autonomy, competence, and self-care agency. We assessed feasibility using quantitative data collected based on a single group repeated-measures design, along with qualitative data such as patients’ feedback on satisfaction during patient counseling. The program comprised video education and individual counseling by nurses. Thirty patients completed this program. Outcome variables were measured thrice: before education, immediately following the first week of video education, and after two consecutive weeks of counseling. A repeated measures ANOVA showed a statistically significant increase in autonomy (F = 5.03, *p* = .038), competence (F = 17.59, *p <* .001), and self-care agency (F = 24.19, *p <* .001). Our pilot study provided preliminary evidence supporting the feasibility for implementation of the theory-based self-management program, and suggesting its preliminary effects in improving autonomy, competence, and self-care agency among kidney transplant recipients. Further research is needed to examine the short- and long-term effects of this program in a longitudinal, randomized control study with a larger sample.

## Introduction

Kidney transplantation, when compared to dialysis, improves the survival rate [1, 2] and quality of life [3, 4] of patients with chronic renal failure. The number of annual transplants is increasing worldwide [5], and Korea is no exception [6]. After kidney transplantation, patients are required to take immunosuppressants for life and perform self-care activities to prevent the side effects associated with these drugs. However, due to immune function deterioration [7–9], transplant patients may experience complications such as an increased risk of infection or cancer, which has been reported to negatively affect their quality of life [10]. Therefore, it is vital that patients were aware of the importance of treatment adherence and continued self-management [11].

There are two types of kidney transplantation. When the transplant occurs from a living donor, the surgery is scheduled in advance, and therefore, self-management education can be planned. However, when using a cadaver donor, patients may undergo surgery suddenly, which may not leave enough time for the provision of appropriate education beforehand [7]. Additionally, cognitive and memory loss due to pre-transplant chronic disease, surgery, or anesthesia decreases educational effectiveness, and a single educational session may not meet patients’ needs [7, 11]. Therefore, regardless of the transplant mode, systematic self-management education should be provided to patients through professional education and continuous self-learning.

Previous research on kidney transplant education has demonstrated that patients prefer to receive complete education about self-care activities before being discharged [11–14]. Generally, studies reported that patients were satisfied with their education on medications and managing side effects, but complained about the lack of education on emotional management and specific self-care activities [11, 13]. Patients also wanted interactive education in addition to printed booklets, and preferred individualized education over group education [12, 14].

In hospitals selected for this study, education on self-management after transplantation was offered to patients in one session before discharge by a staff nurse or coordinator. This discharge education was provided through a booklet and lasted one hour. Since this educational method presented extensive content within a short time, the effect on patients was low due to increased fatigue and decreased concentration. This education method was also not suitable for patients with hearing or visual impairment. To create better educational opportunities, a self-care management program was developed based on self-determination theory (SDT).

Results of previous studies based on the SDT have shown that perceived autonomy support from health providers (henceforth referred to as “autonomy support”) and competence significantly influenced self-care behavior [15–17]. A meta-analysis suggested that intervention programs designed to support autonomy were more effective when they included both reading materials and electronic media [17]. Therefore, it is important to develop a theory-based self-management program that combines video education and nurse-led individual counseling to increase patients’ perceived autonomy support and competence. This study aimed to explore the feasibility and preliminary effects of this program.

## Materials and methods

### Research design and sample

This study used a repeated measures design to examine the feasibility and preliminary effects of a theory-based self-management program for kidney transplant patients. Participants of this study were at least 18 years of age and they were admitted to the kidney transport ward of a university hospital in G Metropolitan City, South Korea. The educational program was implemented from August 6, 2018 to April 30, 2019. During this time, 32 patients were hospitalized for kidney transplantation. The study was described to all patients, following which, they expressed their intention to participate and voluntarily provided written informed consent. Though 32 patients were enrolled in the program, two patients died of complications after transplant surgery, while the remaining 30 patients completed the program.

### Measures

#### Autonomy support

The Health Care Climate Questionnaire, developed by Williams et al. [18] and modified by Seo and Choi [16], was used to measure autonomy support. This tool comprises 15 items assessed on a 7-point Likert scale ranging from 1 (strongly disagree) to 7 (strongly agree), with higher scores indicating higher perceived autonomy support. The Cronbach’s alpha coefficient was .89 in the original study [18], .84 in the study conducted by Seo and Choi [16], and .96 in the present study.

#### Competence

A basic psychological needs scale, originally developed by Deci and Ryan [19], translated into Korean by Lee and Kim [20], and modified by Jeong and So [15], was used to measure competence. This tool comprises six items assessed on a 7-point Likert scale ranging from 1 (strongly disagree) to 7 (strongly agree), with higher scores indicating higher competence. The Cronbach’s alpha coefficient was .80 in the original study [19], .88 in that by Jeong and So [15], and .90 in the current study.

#### Self-care agency

The Self-as-Carer Inventory developed by Geden and Taylor [21], translated into Korean by So [22], and modified by Kim and Suh [23], was used to measure self-care agency. This tool comprises 33 items across the following 6 subscales: cognitive (11 items), physical skills (8 items), decision-making and processing (5 items), information seeking behavior (4 items), awareness of self-regulation (one item), and attention to self-management (4 items). Each item is assessed on a 6-point Likert scale ranging from 1 (strongly disagree) to 6 (strongly agree), with higher scores indicating higher self-care agency. The Cronbach’s alpha coefficient was .96 in the study by Geden and Taylor [21], .96 in that by Kim and Suh [23], and .85 in the present study.

### Procedures

**Fig 1:**
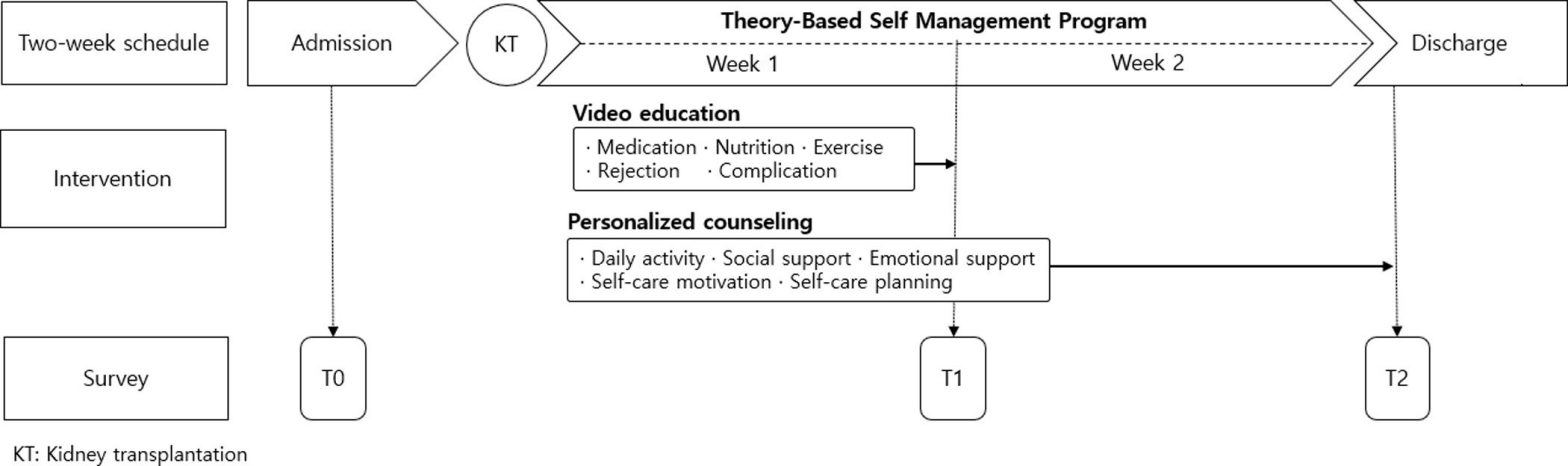
Study design.

The study procedure involved the development and implementation of a theory-based self-management program, following which, its preliminary effects were assessed (Fig 1).

#### Developing a theory-based self-management program

The theoretical framework for this study was based on the SDT, according to which, all behaviors aim to achieve need satisfaction [24]. Intrinsic motivation is the most autonomous form of motivation. It concerns active engagement with tasks that individuals find interesting, which in turn promote growth. Intrinsically motivated behaviors are based on individuals’ need to feel competent and self-determined [19].

In this study, a self-management program was developed to create autonomy in a supportive climate and to achieve competence in self-management behavior after undergoing kidney transplant (Table 1). This two-week program included video education and nurse-led personalized counseling.

**Table 1:** Major concepts of the self-determination theory and its application to the current program.

Concepts	Definition	Application to the Program
Health Care Climate • Autonomy-supporting climate	An atmosphere that encourages individuals to engage in health behaviors for their own reasons, facilitates them to deal with barriers to change, and encourages them to achieve self-respect and acceptance.	Provides a clear and meaningful rationale for self-care activities; acknowledges the importance of a patient’s feelings and agenda; provides options for patients to choose preferred activities.
Satisfaction of Basic Psychological Needs • Competence	A feeling of being effective in producing desired health outcomes and exercising individual capacities.	Encourages the process of setting of realistic goals and discussing expectations of self-care outcomes; provides positive and relevant informational feedback.
Outcome • Self-care agency	An integrated learning capability for performing self-care activities.	Encourages learning about self-care activities through video clips and personalized counseling; Engages patients’ support system, including primary caregiver(s).

An autonomy-supportive climate refers to a caring atmosphere that encourages individuals to engage in healthy behaviors for personal gain, facilitates success in dealing with barriers to change, and helps establish feelings of acceptance and self-respect [25]. Nurses acknowledged the importance of patients’ feelings and agendas, provided clear and meaningful rationales for self-care activities, and offered them relevant options to choose from [26].

Competence is defined as the feeling of being effective in producing desired health outcomes and exercising individual capabilities [25]. Video education was developed along with nurse-led counseling, which taught patients to set realistic goals and discuss expectations of self-care. Additionally, clear and relevant informational feedback was provided after watching the video, followed by the provision of appropriate guidance and support [26]. Contents for the video education were selected based on a literature review on self-care activity. Major themes included medication, nutrition, physical activity, rejection, complications, and daily activity after discharge. The validity of the content was verified by two nurses working in the vascular surgery ward, two organ transplantation coordinators, two professors of nursing, and two professors of transplantation from the vascular surgery department. The content validity index was .97. Five video clips were developed to cover the major themes.

Before the consultation, a nurse created a comfortable atmosphere to encourage patients to express their feelings and experiences about educational contents. The nurse listened to and concentrated on what they said, assuming a non-directive and non-conditional positive attitude. Open-ended questions were used to identify the patients’ understanding of the education and difficulties in compliance with self-care behaviors. Nurse-led patient-tailored counseling was performed using person-centered counseling principles, which encourage changes in self-behavior. Counselors included a professional with a Ph.D. in nursing and a clinical nurse specialist with over three years of experience in caring for kidney transplant patients. Personalized counseling on social and emotional support, daily activity, and self-care plans was provided until patients were discharged.

#### Application and evaluation of the program

Before the commencement of program implementation, researchers explained the purpose and procedure of this study to the research assistant. After a patient was admitted to the ward for kidney transplantation, the research assistant visited the patient and informed him/her of the study purpose and methods. Patients then voluntarily signed a written consent form. Thereafter, a 10–15-minute survey was conducted to collect baseline data.

Intervention began the day the patient was transferred to the ward after kidney transplantation. When participants arrived in the ward, the research assistant gave each patient and their caregiver an orientation on the self-management program. On Days 1–7 in the ward, patients were allowed to watch videos each topic daily using tablet PC or Medical on Demand TV, which was a personal TV attached to the patient bed. After watching a video, nurse-led personalized counseling was conducted for about 30 minutes. Personalized counseling took place in the patient’s room and nurses counseled 3–4 patients a day. Nurses first identified educational needs according to each patient’s condition. During the video education session, nurses educated and counseled patients about the necessity and importance of self-management, including medication, nutrition, physical activities, etc. After watching a video and prior to counseling, participants were asked whether they were satisfied with the video education or not, following which they provided feedback or comments on how the education could be improved. Subsequently, counseling sessions, which were personalized according to age, sex, family, comorbidity, and type of transplant, were conducted until the day of discharge. Nurses identified each patient’s life patterns before transplant surgery took place and motivated them to perform appropriate self-management. The patient’s support system was identified and family was counseled as to their role in the patient’s self-care. Emotional support was also offered. Patients discussed difficulties faced during the transplant waiting period and expressed gratitude for their donors. During counseling, nurses listened to and empathized with patients. Evaluation of the program was conducted after video education and before discharge.

### Treatment fidelity data

Treatment fidelity was assessed focusing on study design, treatment delivery, and treatment receipt [27]. In this study, to ensure treatment fidelity, the intervention was developed based on the SDT and it was reviewed by a panel of experts on the theory, self-management, and kidney transplant. Specifically, to ensure consistency of intervention delivery, the expert panel reviewed the structured video education and semi-structured counseling procedures developed. The video education, including filming a video (e.g., exercise), was conducted by 2 members of the hospital staff working at the department of information technology, with an experience of 5 or more years. The cost of video production was covered by the hospital. To assess treatment delivery, frequency of intervention use (i.e., participation in video education) was examined based on the rent log sheet for the tablet PC used to administer the video education. Treatment receipt was assured by observing patients’ demonstration of exercise performance during counseling.

### Data analysis

Collected data were analyzed using the Statistical Package for the Social Sciences (SPSS) for Windows, version 24. Frequency, percentage, mean, and standard deviation were used to describe patients’ general and transplant-specific characteristics. A repeated measures ANOVA with within-subject factors was performed to assess the effect of the program. First, Mauchly’s spherical test was performed; if the p value was < .05, the Greenhous-Geisser effect test was performed. Post-hoc analysis was performed using the Bonferroni test to compare mean differences between measurements. A p value of < .05 was considered statistically significant. Cohen’s dz [28] was used to calculate the effect size for the video education program and a partial eta squared was used to compute the effect size for the entire program.

### Ethical considerations

This study was conducted after approval from the Institutional Review Board (CNUH-2018-140) of the university hospital in Gwangju, South Korea, where the current study was conducted. Participants were informed about the purpose of the study, and their rights to anonymity, confidentiality, and freedom to withdraw from the study. Written informed consent was obtained from those who wished to participate in the study.

## Results

### General and transplant-related characteristics

The mean age of participants was 49.10±10.01 years and 18 of them were male (60.0%). Further, 17 (56.7%) participants were married, 14 (46.6%) were college graduates, and 15 (50.0%) were employed.

Of all participants, 16 (53.3%) received a transplant from cadaver donors and 26 (86.7%) had undergone dialysis before transplantation. The mean period of dialysis was 5.23±3.74 years and the mean period of kidney failure was 7.50±6.59 years.

### Difference in self-care agency according to general and transplant-related characteristics

Before the transplant, married patients demonstrated significantly higher self-care agency than did single patients (t = -3.09, *p* = .004). However, there were no statistically significant differences in self-care agency scores after video education and before discharge. Similarly, at all three measurement points, self-care agency scores did not differ significantly with reference to gender, age, education level, monthly income, and religion.

Significant difference was seen in self-care agency scores according to the type of donor. Self-care agency scores for patients who underwent transplantation from a deceased donor were significantly lower than scores for patients undergoing living donor transplantation in all measurements (t = -2.46, *p* = .020; t = -2.74, *p* = .011; t = -3.57, *p* = .001). There was also no statistically significant difference in self-care agency scores according to dialysis before transplantation. The durations of dialysis and renal failure were not statistically significant when correlated with self-care agency scores (Table 2).

**Table 2:** Differences in self-care agency by general and transplant-related characteristics (N = 30).

Characteristics	Categories	n (%) or M (SD)	Self-care agency					
			T0		T1		T2	
			M (SD)	t/F/r (*p*)	M (SD)	t/F/r (*p*)	M (SD)	t/F/r (*p*)
Sex	Men	18 (60.0)	158.78 (23.57)	0.77	168.06 (20.50)	0.13	175.67 (17.96)	0.46
	Women	12 (40.0)	152.25 (21.24)	(.446)	167.08 (20.65)	(.900)	172.58 (18.02)	(.650)
Age (years)	≤30s	5 (16.7)	155.80 (24.75)	0.83	167.60 (23.55)	1.74	179.20 (22.02)	1.08
	40s	11 (36.7)	150.36 (22.92)	(.490)	157.82 (22.04)	(.183)	166.82 (16.02)	(.376)
	50s	7 (23.3)	154.29 (22.90)		173.14 (17.17)		178.57 (19.03)	
	≥60s	7 (23.3)	167.43 (20.74)		177.71 (12.98)		178.86 (15.49)	
Marital Status	Single	13 (43.3)	143.38 (19.83)	-3.09	160.54 (21.73)	-1.75	174.08 (18.46)	-.10
	Married	17 (56.7)	165.94 (19.76)	(.004)	173.12 (17.69)	(.091)	174.71 (17.74)	(.925)
Education	Below middle school	5 (16.7)	162.20 (26.76)	0.40	177.40 (16.23)	1.30	178.60 (17.86)	1.26
	High school	11 (36.7)	151.73 (18.71)	(.674)	160.82 (22.34)	(.289)	167.73 (17.86)	(.299)
	College and over	14 (46.6)	157.50 (24.69)		169.57 (19.13)		178.21 (17.28)	
Employment status	Employed	15 (50.0)	162.13 (26.34	1.48	171.20 (19.77)	0.96	179.00 (17.79)	1.44
	Unemployed	15 (50.0)	150.20 (16.76)	(.150)	164.13 (20.69)	(.347)	169.87 (17.05)	(.162)
Monthly Income (1,000\)	Less than 100	5 (16.7)	154.40 (12.34)	1.15	165.80 (24.95)	0.76	174.00 (21.25)	1.15
	100–300	9 (30.0)	148.56 (26.11)	(.349)	163.00 (19.24)	(.530)	168.78 (16.07)	(.349)
	More than 300	10 (33.3)	166.50 (21.06)		175.50 (14.30)		182.50 (15.74)	
	No response	6 (20.0)	151.83 (24.24)		163.17 (26.84)		169.83 (19.79)	
Religion	Protestant	7 (23.3)	157.00 (25.22)	0.25	163.43 (24.33)	2.44	171.71 (17.26)	1.66
	Buddhist	5 (16.7)	156.00 (23.23)	(.862)	181.00 (14.71)	(.087)	184.80 (15.09)	(.199)
	Catholic	4 (13.3)	164.75 (16.58)		182.75 (10.37)		185.25 (16.96)	
	No religion	14 (46.7)	153.36 (24.02)		160.71 (18.60)		169.00 (17.75)	
Type of Donor	Cadaver	16 (53.3)	147.44 (23.01)	-2.46	159.13 (20.13)	-2.74	165.31 (13.74)	-3.57
	Living	14 (46.7)	166.14 (17.89)	(.020)	177.43 (15.84)	(.011)	184.86 (16.28)	(.001)
Dialysis before transplantation	Yes	26 (86.7)	155.69 (22.30)	-0.29	169.15 (18.70)	1.02	175.65 (17.51)	0.96
	No	4 (13.3)	159.25 (27.27)	(.744)	158.00 (29.82)	(.312)	166.50 (19.77)	(.346)
Duration of dialysis (yrs) (n = 27)		5.23 (3.74)	156.17 (22.52)	-.240 (.228)	167.67 (20.21)	-.134 (.504)	174.43 (17.74)	-.140 (.486)
Duration of CKD (yrs)		7.50 (6.59)		.178 (.347)		.168 (.375)		.154 (.418)

*Note*. KT = Kidney transplantation; CKD = Chronic Kidney Disease.

### Preliminary effects of the self-management program

A repeated measures ANOVA was used to assess the preliminary effects of the theory-based self-management program. According to Mauchly’s test, no variable met the assumption of sphericity. Therefore, tests of within-subject effects were performed using the Greenhous-Geisser test (Table 3). The autonomy support score differed significantly across measurement points (F = 5.03, *p* = .038; Cohen’s dz = 0.50; partial eta squared = 0.15). Specifically, after video education, the autonomy support score increased significantly by 4.54 points as compared to prior scores (*p* = .038), and these scores tended to continue to increase until discharge.

**Table 3:** Effects of the self-management program on autonomy support, competence, and self-care agency (N = 30).

Variables	T0^a^		T1^b^		T2^c^		F	*p*	ES (T0-T1)	ES
	Mean	SD	Mean	SD	Mean	SD			Cohen’s dz	η_p_²
Autonomy Support	90.53	12.68	95.07	9.10	95.37	10.01	5.03	.038 (a<b)	0.50	0.15
Competence	29.73	7.79	33.93	6.10	35.90	5.38	17.59	< .001 (a<b<c)	0.73	0.38
Self-care Agency	156.17	22.52	167.67	20.21	174.43	17.74	24.19	< .001 (a<b<c)	0.75	0.46

*Note*. T0 = before the operation; T1 = after video education; T2 = before discharge; ES = effect size.

Similarly, the competence score differed significantly across measurement points (F = 17.59, *p <* .001; Cohen’s dz = 0.73; partial eta squared = 0.38). Specifically, after video education, the competence score increased significantly by 4.20 points as compared to prior scores (*p* = .001), and by 6.17 points prior to discharge (*p <* .001). Thus, competence tended to continue to increase until discharge.

The self-care agency score also differed significantly across measurement points (F = 24.19, *p <* .001; Cohen’s dz = 0.75; partial eta squared = 0.46). After video education, the score increased significantly by 11.50 points a compared to prior scores (*p* = .001), and by 18.27 points prior to discharge (*p <* .001). Again, self-care agency tended to continue to increase over time.

### Treatment fidelity of the intervention

Findings of the treatment fidelity assessment have been summarized in Table 4. It was observed that the intervention was implemented, as intended, according to the study design, and that it supported treatment delivery and receipt. Approximately 93.8% patients participated in the video education and personal counseling at least once. The top two most-frequently asked questions during counseling pertained to information needs for allowed or prohibited foods (56.3%) and return to work, housework, and/or social life including travel, physical activity, and sex life (25.0%). When they were asked about satisfaction, feedback, and comments on the intervention, all answered that they were satisfied, and some of them provided feedback such as, *“I prefer video clips or multimedia than text because I don’t know much about the text and my eyes get darker as I get older*.*”*, *“I hope you can confirm my exercise posture*.*” and “I struggled to find speaker icon to increase sound volume*.*”*

**Table 4:** Treatment fidelity assessment of the theory-based self-management program.

Treatment fidelity component	Method	Intervention group
Study design	Use of the intervention based on the self-determination theory	The program was developed and reviewed by the expert panel
Treatment delivery	Video education installed in Medical on Demand TV* or tablet PC	The MP4 format of educational material was provided using Medical on Demand TV or tablet PC
	Tablet PC rent log	93.8%
	Counseling attendance log	93.8%
Treatment receipt	Observation of the patient’s demonstration of exercise performance	After watching the education, all patients performed exercise adequately.

* Tracking data on Medical on Demand TV (personal TV attached to the patient bed) was not available.

## Discussion

This study examined the feasibility and preliminary effects of the theory-based self-management program developed for kidney transplant patients. This program was in operation for eight months on a transplant ward. At the time of the study’s announcement, all transplant patients were interested in participation. All patients who underwent transplants during the study period were enrolled in the program. Thirty patients completed the two-week program, except for two patients who died during hospitalization (retention rate, 93.8%). The program consisted of five sessions, in one week, of video education on topics including medication, nutrition, physical activity, graft rejection, complications, and daily activity, followed by nurse-led individual counseling for two weeks.

As a result of this study, participants’ agency in self-care improved significantly over time. The effect size of the video education was large, as was the effect size of the program as a whole [29]. Looking at programs developed to improve self-care agency, nurse-led educational programs have been considered effect [23, 30] and results of this study are consistent. The merits of this program were that both supportive counseling by nurses conducted daily for two weeks and video education of less than three minutes were found to be very effective in improving participants’ agency in self-care. According to a study of trajectories of self-care agency in lung transplant recipients over the first twelve months following transplantation [31], those demonstrating high self-care agency just before discharge maintained high levels through the first year after transplant. Further research is needed to understand the ongoing effects of this program and the degree of change in actual self-care behavior.

This study showed that patient competence was significantly improved over time, as the effect size of both video education and the program as a whole were medium [29]. Competence is the subjective perception of one’s ability to interact effectively with their environment [19] and is similar to the concept of self-efficacy [16]. In this study, competence was measured by achievement and confidence in self-care activities after kidney transplantation. Denny et al. reported a significant five-minute educational video intervention on stroke that significantly improved knowledge about stroke and self-efficacy in recognizing symptoms [32]. Davis et al. also reported that self-efficacy was improved for glaucoma patients after watching a four-minute educational video [33]. Three-minute video education in this program was formally provided to patients, consisting of one topic per day for five days. After regular video education, patients were free to watch the video at any time. Nurse-led individual counseling sessions were used to provide a clear rationale for self-care activities and to offer relevant informational feedback. Participants were regarded as having gained efficacy in self-management by solving questions presented through video education and nurse-led personalized counseling. However, according to a review of video-based educational interventions in hospital settings [34], while such interventions were shown to improve short-term health literacy goals, the long-term impact on behavior or lifestyle modifications was unclear. Therefore, educational programs linked to follow-up management are needed so that agency in self-care can be maintained after discharge, thus strengthening self-management behavior.

Results of this program showed that participants’ autonomy support scores improved significantly after video education and maintained constant levels through time of discharge. The effect size of video education was medium and the effect size of the program itself was small [29]. Autonomy support is an important variable in improving self-care behavior in patients with chronic diseases requiring long-term self-care. According to a study analyzing differences in high risk factors, autonomy support, and health behaviors between relapsed and non-relapsed patients more than a year after coronary intervention [35], autonomy support was a significant predictor for relapse in patients with coronary artery disease, and patients with low autonomy support were approximately four times more likely to relapse than patients with high autonomy support. The predictive model of self-management behavior for patients with type 2 diabetes showed that autonomy support influences self-management behavior through mediation of autonomous motivation and competence [16]. A study of structural equation modeling in self-care behaviors of kidney transplant patients showed that autonomy support influenced self-care behavior by mediating basic psychological needs, including competence [15]. In the case of this program, nurses attempted to offer sufficient information using non-controlling language during counseling to form an atmosphere in which patients’ could determine self-management behaviors effectively. These activities were believed to increase autonomy support for participants. However, the effect size of this support in the program as a whole is low, and the self-management program needs revision in order to improve patient’s autonomy support in the future.

In the present study, the mean score of single participants’ self-care agency was significantly lower than that of married participants before transplantation, but there was little difference two weeks after the transplant was performed. Self-care behavior is affected by one’s social network, as well as by their own agency regarding self-care [36]. Social support from caregivers or family members at all times during hospitalization seemed to offer the largest impact on improvement in self-care agency scores of single patients after transplantation. Therefore, it is necessary to involve the social support system of patients in self-management programs developed for future study.

The results of this study indicate that scores for self-care agency were significantly different at three measurement points according to the type of donor. Patients undergoing living donor transplantation had significantly higher self-care agency scores than patients undergoing deceased donor transplantation. This can be explained in part by differences in the procedures of transplantation. In the case of a living donor transplantation, patients are systematically educated and consulted by the coordinator. In contrast, patients receiving transplants from deceased donors are often not properly educated due to the abrupt nature of this surgery. This lack may make a huge difference in self-care agency scores between the two groups. A familial relationship between the recipient and donor may also explain this difference. All participants in this study undergoing live transplants received a kidney from a family member. SDT suggests that if basic psychological needs, such as relatedness, are satisfied, autonomous motivation is improved and related behaviors can be maintained [19]. The study of factors affecting self-management behavior of liver transplant patients also reported that family relatedness influenced a change in health behaviors of participants [37]. Patients who received kidney transplants from a family member were likely to appreciate the sacrifice and demonstrated more autonomous motivation to protect their kidneys. Based on the results of this study, we recognize the necessity of exploratory studies on kidney transplant experiences for patients according to the type of donor and differentiated intervention strategies necessary for patients undergoing deceased donor transplantation to improve self-care agency.

Patients were active during the program and showed high interest in self-management. Most of participants expressed high satisfaction in all the video clips and individual counseling. Especially, in the case of illiterate patients over 60 years of age, education using existing booklets had limitation in improving patient understanding, but the degree of satisfaction was greatly increased through various audiovisual materials and nurse-led counseling of this program. The most common question asked by patients concerned diet. Patients had a desire to limit food intake during periods of dialysis. Patients also wanted to know to what extent they could return to work, social life, housework, etc. Few showed anxiety about the possibility of transplant failure. Based on this preliminary study, we recognize the need to further elaborate the contents of customized dietary education and daily activities for transplant patients.

This study has some limitations. First, the major limitations of this study were its small sample size, absence of a control group, and the short follow-up period. These issues limit the generalizability of the present findings. Therefore, further research should be conducted with larger samples, including a control group, and they should examine the long-term effects of the intervention by conducting a longitudinal randomized controlled trial (i.e., three months and one year). Further, studies should include not only self-management but also graft survival, and adherence to clinical blood labs monitoring and routine follow-ups. Second, the present findings may reflect a response bias as participants could provide socially acceptable answers to researchers because they may feel pressurized by a Likert scale survey. Third, an attention bias may have occurred owing to the study setting. This study was conducted at one university hospital, which has approximately 1,300 beds and an average of 30 to 40 kidney transplants per year. Because the hospital follows a two-week hospitalization protocol from admission to discharge, participants may receive intensive attention during the hospitalization. Therefore, the present findings should be interpreted with caution.

## Conclusion

Despite these limitations, our pilot study provides preliminary evidence supporting the feasibility of implementation of a nurse-led theory-based self-management program, suggesting its preliminary effects in improving autonomy, competence, and self-care agency among kidney-transplant recipients. Ongoing work in the field is vital to prevent transplant-related side effects and complications and to maintain and/or improve physical function and emotional status, through appropriate self-care management education, in kidney-transplant patients.

## Supporting information

S1 File Contents of the video education in the self-management program for kidney transplant patient. (DOCX)

S2 File Dataset. (SAV)
