# Supplementary material for: Feasibility and preliminary effects of a theory-based self-management program for kidney transplant recipients: A pilot study
Source: PLoS One. 2021 Jun 30;16(6):e0248947. doi: 10.1371/journal.pone.0248947 (PMC8244880; doi:10.1371/journal.pone.0248947)
Supplement: S1 File — (DOCX) [file pone.0248947.s001.docx]

S1 File. Contents of the Video Education in the Self-Management Program for Kidney Transplant Patients

| Topics | Contents | Running  Time  (min) | Screenshots | |
| --- | --- | --- | --- | --- |
| Medication | -Type of transplant medications  -How to take drugs  -Side effects of drugs  -How to prevent side effects of drugs | 2:35 | 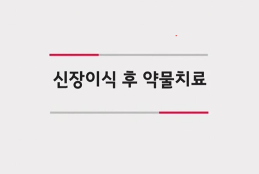 | 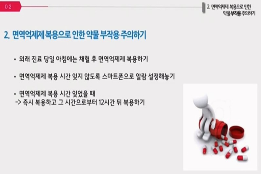 |
| Nutrition | -Maintaining a healthy weight  -Healthy diet  -Diet restrictions  -Daily fluid intake | 2:24 | 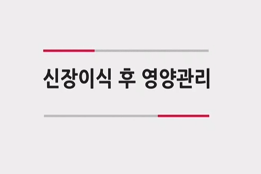 | 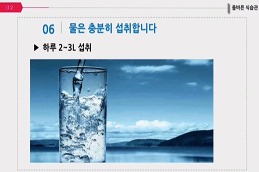 |
| Exercise | -Why exercise is important  -Type of exercise  -Frequency of exercise  -Exercise precautions | 3:50 | 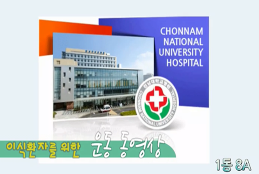 | 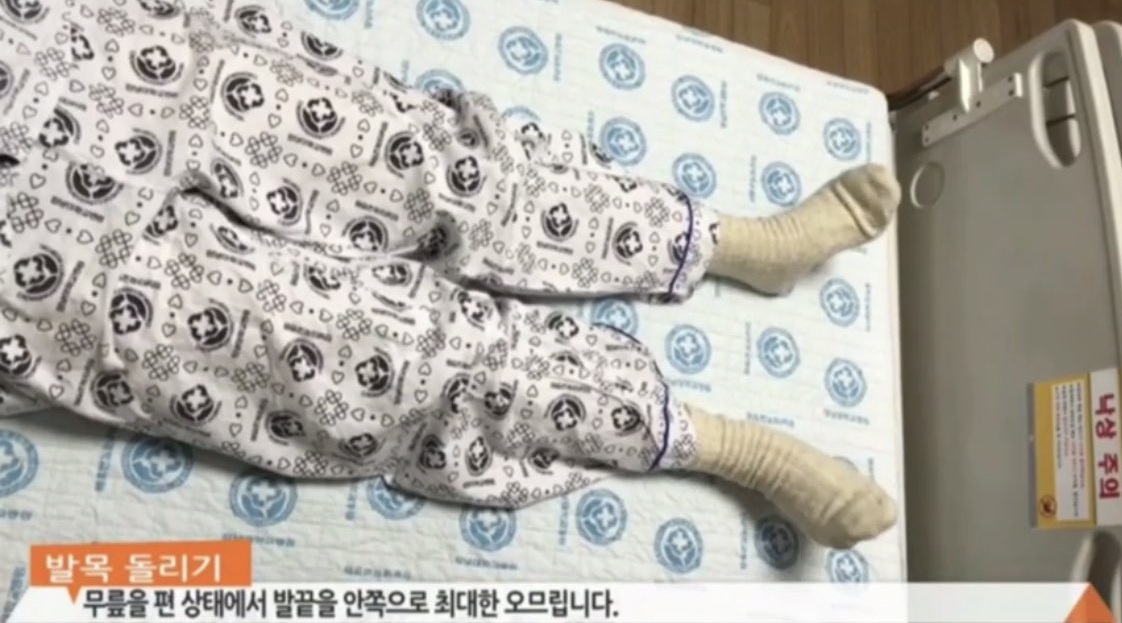 |
| Rejection & Complication | -Types of rejection  -Signs and symptoms  -Treatments  -Preventing rejection and complications | 3:54 | 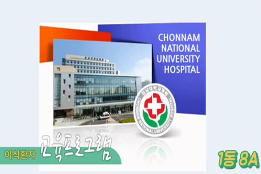 | 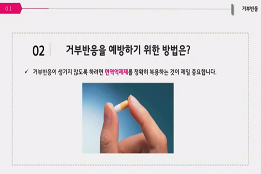 |
| Daily activity | -Infection precautions  -Sex life  -Physical activity  -Work or school life  -Travel or going out  -Regular outpatient visits | 3:12 | 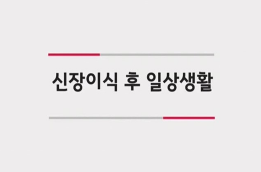 | 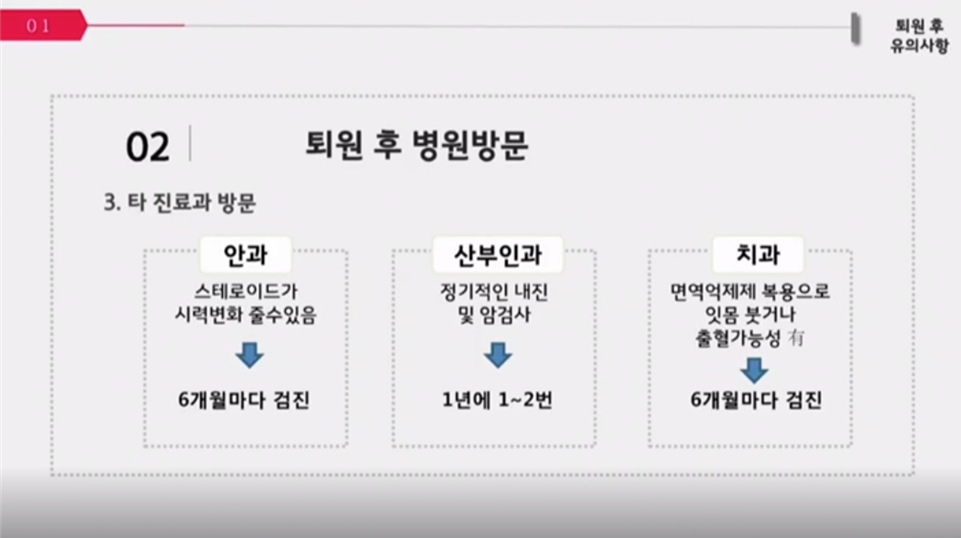 |
